# Comprehensive determinants of health service utilisation for mental health reasons in a canadian catchment area

**DOI:** 10.1186/1475-9276-11-20

**Published:** 2012-04-02

**Authors:** Marie-Josée Fleury, Guy Grenier, Jean-Marie Bamvita, Michel Perreault, Yan Kestens, Jean Caron

**Affiliations:** 1 Department of Psychiatry, McGill University, Douglas Hospital Research Centre, 6875 LaSalle Blvd., Montreal, Quebec H4H 1R3, Canada; 2 Douglas Hospital Research Centre, 6875 LaSalle Blvd., Montreal, Quebec H4H 1R3, Canada; 3 Department of Social and Preventive Medicine, University of Montreal, 3850, St-Urbain Street, Montreal, Quebec H2W 1T7, Canada

**Keywords:** Mental disorders, Emotional problems, Longstanding violence victimisation, Neighbourhood, Health service utilization

## Abstract

**Introduction:**

This study sought to identify factors associated with health service utilisation by individuals with mental disorders in a Canadian catchment area.

**Methods:**

To be included in the study, participants had to be aged between 15 and 65 and reside in the study location. Data was collected randomly from June to December 2009 by specially trained interviewers. A comprehensive set of variables (including geospatial factors) was studied using the Andersen's behavioural health service model. Univariate, bivariate, and multivariate analyses were carried out.

**Results:**

Among 406 individuals diagnosed with mental disorders, 212 reported using a mental health service at least once in the 12 months preceding the interviews. Emotional problems and a history of violence victimisation were most strongly associated with such utilisation. Participants who were middle-aged or deemed their mental health to be poor were also more likely to seek mental healthcare. Individuals living in neighbourhoods where rental accommodations were the norm used significantly fewer health services than individuals residing in neighbourhoods where homeownership was preponderant; males were also less likely to use services than females.

**Conclusions:**

Our study broke new ground by uncovering the impact of longstanding violence victimisation, and the proportion of homeownership on mental health service utilisation among this population. It also confirmed the prominence of some variables (gender, age, emotional problems and self-perceived mental health) as key enabling variables of health-seeking. There should be better promotion of strategies designed to change the attitudes of males and youths and to deal with violence victimisation. There is also a need for initiatives that are targeted to neighbourhoods where there is more rental housing.

## 

Mental disorders are among the leading causes of morbidity worldwide. In Canada and the United States, depression is the primary source of occupational disability [1-3]. Improving the efficiency of the mental healthcare system, particularly service utilisation, is a priority. According to epidemiological studies, an exceedingly high proportion of individuals with mental disorders do not seek professional help despite the availability of effective treatment [4-9]. A recent meta-analysis of 27 studies found that 26% of Europeans sought healthcare for a mental disorder in a 12-month period [10]. Another study, comparing 17 countries, revealed that, in the 10 countries with the highest per-capita income, between 13% and 61% of individuals, depending on the severity of their mental disorders, received health services [11,12]. According to the 2002 Canadian Community Health Survey of Mental Health and Well-Being (CCHS 1.2), only 39% of Canadians used services for mental health reasons [13]. These findings suggest that mental health systems must identify individuals who need care more effectively, and remove clinical and societal barriers to health services.

The most frequently employed tool for identifying factors associated with health service use is the Behavioural Model of Health Service Use [14]. It has been applied to health surveys [15-18] of the general population and mental health studies [9,19-22]. It has also served in studies of older patients with psychiatric problems [23-26], patients with severe mental disorders [27-29], and patients with concurrent problems such as homelessness, substance abuse or violence [30-33].

The Behavioural Model of Health Service Use encompasses individual and contextual dimensions. It classifies predictors of service use into three categories: predisposing, enabling, and needs-related factors. Predisposing factors are individual characteristics that exist prior to the illness (for example, socio-demographic profile, attitudes and values, and knowledge about services). Enabling factors refer to various features that influence care delivery and attitudes toward care; they encompass variables such as income, social support, and availability of care. Finally, needs-related factors include physical and mental health assessment by patients and professionals, for example, illness, symptom severity, perceived needs, and impairment requiring services.

In general, needs-related factors are most closely associated with health service utilisation. Reportedly, several needs-related factors have significant effects on the use of health services: diagnoses of schizophrenia, serious depression [34], and social anxiety behaviour [34].

Among predisposing factors, age, gender, marital status, education, country of birth, and race/ethnicity are significant determinants of service utilisation among individuals with mental disorders. Several studies have found that younger (18-24) and older (65 and up) individuals were less likely to use services than participants aged 25 to 64 [8,34,35]. Females were the most frequent users of health services, principally of general practitioners; men were more likely to seek specialised services [2,29,34,36-39]. Studies have found that persons who were previously or currently married used services more often than bachelors [2,8,40,41]. Individuals with more education [2,34] also used health services significantly more often than less-educated persons, despite the higher incidence of mental disorder among the latter [42]. With regard to country of birth, race and ethnicity, studies found that Whites are more likely to use health services than Blacks or immigrants [2,30,33,43]. Attitudes and beliefs regarding mental health and treatment and knowledge also played a role in service use. High rates of health service use have been found among individuals who consider their mental health to be poor [34] and maternal history of mental illness [5].

Among enabling factors, perceived barriers to accessibility to care were negatively associated with service utilisation [34]. Individuals with more elevated socio-economic status tended to use specialised services more assiduously, particularly psychiatric and psychological care, even among individuals with the same insurance coverage [2,44-46]. Family and social support can be positively or negatively associated with service utilisation [29,47,48]. Some social networks helped individuals to recognise their problems and seek aid from health providers; other networks tended not to encourage members to seek help, thereby constituting a barrier to accessibility [49]. Professional support also played a role [31,50]. Access to a regular source of medical care was positively associated with service utilisation [34].

If previous studies have found several variables associated with health service utilisation for mental health reasons, some variables such as religious beliefs, neighbourhood/geospatial information, involvement in the justice system, impulsivity, and lifetime of aggressive behavior and violence, have received little or no attention in the literature to our knowledge. Some studies have found that churches were a significant factor of social integration in certain communities, and that church involvement was negatively associated with depressive symptoms. Concerning neighbourhood/geographical variables, living in socio-economically deprived areas or socially disorganised neighbourhoods was associated with psychosocial stress and higher incidence of depressive symptoms [51]. Finally, past or present experiences of violence, crime or imprisonment were all associated with higher prevalence of mental disorders [52] or emotional problems [53,54]. Those variables that were positively or negatively associated with the prevalence of mental disorders could also play a role in health service utilisation for mental health reasons.

This study sought to identify factors of health service utilisation among individuals diagnosed with a mental disorder in a 12-month period within a Canadian urban catchment area of 258,000 inhabitants. A comprehensive set of variables comprising geospatial factors was analysed using the Andersen's behavioural model [14], which posits that health service use is determined by predisposing, enabling, and needs-related factors. We hypothesised that some novel variables associated with the prevalence of mental disorders in the general population (e.g. neighbourhood or geospatial data, involvement in the justice system), based on a holistic set of variables, could also play a role in health service utilisation.

## Methods

### Study design and setting

This study focused on a catchment area in the south-western sector of Montreal, Canada with a population of 258,000. The area encompasses a diverse mix of residents as well as a broad range of social structures, socio-economic status, education, availability of health services, neighbourhood dynamics, and levels of security.

The study area included six neighbourhoods ranging in population from 29,205 to 72,4200 [55]. Immigrants represented 17% of the population (as compared with 26% in Montreal). The proportion of low-income households was 36% (as compared with 23% in the province of Quebec and 35% in Montreal). Low-income households were located mainly in two of the four neighbourhoods where nearly half of residents are low-income earners. Previous studies revealed a high incidence of psychological distress in low-income populations at this location [55]. Mental health services are chiefly delivered by three organisations: two health and social service centres (created through the merger of a general hospital, community local service centres, and nursing homes) that provide first-line (basic bio-psycho-social services offered to the population with common mental disorders) and second-line (specialized services) health services. A psychiatric hospital delivers second- and third-line services (ultra-specialized for individuals who present highly complex health problems). Sixteen community-based agencies offering mental health services were also present; they provided numerous services (for example, a crisis centre, day centres, self-help groups, back-to-work programs) to individuals with mental disorders or their relatives. General practitioners and psychologists in private clinics completed the mental health offer in this area.

### Selection criteria and sample

To be included in the survey, participants had to be aged from 15 to 65 and be residents of the target area. The objective was to obtain a representative sample of the targeted population, both geographically, i.e. recruiting participants across the area, as well as in proportion to the population density and in terms of socio-economic status (SES), that is, representative of the educational attainment profile of the area. A target sample of 3,708 addresses was selected for recruitment. Participants were first contacted by phone, and 300 individuals were recruited. It was soon decided, however, to proceed otherwise due to the low response rate compared to the number of potential participants who were randomly selected. In order to improve recruitment, 13 nearby addresses were added to each address that was originally selected. This range of 14 potential addresses thus included the original address, the three addresses that were the closest on each side, and the seven addresses on the opposite side of the street. We used 3,408 addresses for door-to-door recruitment; the 3,408 original addresses hence resulted in 3,408*14 nearby addresses, theoretically representing 47,712 potential addresses where individuals would be available for recruitment. The recruiters met 7,265 individuals in person and, from those, 3,726 (51%) refused to participate. Amongst the 3,539 individuals who accepted to participate, 1,405 (19%) were not eligible; 2,134 participants completed the interview for a response rate of 49%. The response rate is superior to the median rates reported in epidemiological studies of populations conducted in the years since 2000, when there had been a steady decline in participation rates over the past 30 years [56,57].

The sample was equally distributed in the study area among the various neighbourhoods. Data was collected randomly from June to December 2009 by specially trained interviewers. Only one person per target household was selected using procedures and criteria contained in the *National Population Health Survey *[58]. For purposes of recruitment, the interviewers had phone contacts with individuals who agreed to participate in the study within the week during which they were recruited, in order to schedule a face-to-face meeting either at the participant's home or in an office designated for that purpose at the psychiatric hospital. Most interviews, however, were conducted at the participant's home. The face-to-face interview was conducted once a consent form was signed, and lasted from a minute and a half to three hours depending on whether a mental disorder was detected or not. Interviewers used portable computers. The research was approved by relevant ethics boards.

A randomly selected sample of 2,434 individuals took part of the survey. Women were overrepresented (62%) in the final study sample, compared to the reference population (52%); men under the age of 45 were underrepresented. To ensure a precise prediction of the prevalence of mental illness in the population, we weighted the data for sex and age. The mean age was 40,72 (ET = 14,08). The make-up of respondents was as follows: 49% were men, 38% were single, 45% were married or in a relationship, 12% were divorced or separated, and 71% had a post-secondary diploma; 79% were employed in the last 12 months and 25% were immigrants; French was the primary language spoken by 54% of the respondents, followed by English as the primary language for 22%; Whites accounted for 81% of the sample. The average personal income was $31,192 CAD (sd-33) and the average family income $59,056 CAD (sd = $49,851); 33% of the respondents were considered as having a low income and 67% had extremely low or no income. Among the 2,434 individuals who took part in the survey, 406 had experienced at least one episode of mental health disorder in the 12 months preceding the interview, and were selected for the analyses described below.

### Variables and measurement instruments

Variables assessed in this study are displayed in Table 1 and categorised according to the behavioural model [14]. Instruments used to measure specific health and psychosocial parameters are described in Table 2.

**Table 1:** Variables assessed in the study

		Variables
**Predisposing factors**	Socio-demographic Variables^i^	Age
		
		Gender
		
		Marital status
		
		Household composition and size
		
		Education
		
		First language
		
		Country of birth
	
	Religious beliefs	Importance attributed to spirituality
		
		Frequency of participation in religious activities
	
	Health beliefs	Quality of life^ii^
		
		Self-perception of mental and physical health^i^
	
	Knowledge	Parental Psychiatric History^iii^
	
	Justice system^i^	History of imprisonment

**Enabling factors**	Economic factors^i^	Income (personal, household; main source)
	
	Territory	Neighbourhood^iv-x^
		
		Neighbourhood characteristics^iv-ix^
	
	Social support^xi^	
	
	Social stigma^xi^	
	
	Geospatial variables^i^	Walking distance to health services
	
		Driving distance to health services
	
		Proportion of rental accommodations
	
		Proportion of individuals who moved a year ago
	
		Unemployment rate among the population aged 25 and up
	
		Active population aged 15 and up
	
		Average household income after taxes
	
		Average household income after taxes
	
		Proportion of recent immigrants

**Needs**	Number of mental disorders^xiii-xvi^	
	
	Lifetime victim of violence^i^	
	
	Lifetime aggressive behaviour^xvi^	
	
	Psychological distress^xvii^	
	
	Impulsiveness^xviii^	
	
	Emotional problems^i^	

**Health service utilisation**	Services are provided in hospitals (including hospitalisation), mental health community centres, rehabilitation centres, private clinics, pharmacies, and in the voluntary sector (e.g. support groups, crisis-line services). Professionals consulted included psychologists, general practitioners, psychiatrists, case managers, toxicologists, nurses, social workers, psychotherapists, pharmacists, other health professionals.	

Measuring instruments are indicated by superscripts - see Table 2

**Table 2:** Measurement instruments

		Name	Description
**Predisposing factors**	i	Canadian Community Health Survey (CCHS) 1.2 (Statistics Canada, 2001)*	Survey questionnaire for socio-demographic characteristics; yes/no and multiple choice questions; Likert and non-Likert scale questions
	
	ii	Satisfaction with Life Domains Scale (SLDS) [59]^a^	20 items; seven-point Likert scale questions; Cronbach alpha: 0.92

	iii	Parental Psychiatric History (PPH) [60]	Measures mental disorders in parents and relatives; yes/no questions

**Enabling factors**	iv	Sense of Community Scale (SCS) [61]	8 items; Cronbach alpha: 0.74
	
	v	Community Participation Scale (CPS) [62]	6 items; yes/no and four-point Likert scale questions; Cronbach alpha: 0.73-0.89
	
	vi	Resident Disempowerment Scale (RDS) [63]	3 items
	
	vii	Sense of Collective Efficacy (SCE) [64]	Evaluate the effect of social and institutional mechanisms on individuals living in the neighbourhood; Cronbach alpha: 0.80-0.91
	
	viii	Neighbourhood Disorder Scale (NDS) [63]	9 items; Cronbach alpha: 0.84
	
	ix	Physical Conditions of the Neighbourhood (PCN) [61]	7 items; Cronbach alpha: 0.87
	
	x	Facility in Neighbourhood (FN) [65]	13 items; ten-point Likert scale questions; Cronbach alpha: 0.40 to 0.90
	
	xi	Social Provisions Scale (SPS) [66]^a^	24 items; four-point Likert scale questions; Cronbach alpha: 0.92
	
	xii	Devaluation-Discrimination Scale (DDS) [67]	12 items; six-point Likert scale questions; Cronbach alpha: 0.68-0.99

**Needs**	xiii	Composite International Diagnostic Interview (CIDI), (Statistics Canada, 2000)*	Screening of mental disorders; was used in the World Mental Health, 2000 [68]; included the most frequent mental disorders (depression, bipolar disorder, mania, post-traumatic stress disorder, anxiety disorders: social phobia, agoraphobia, and panic disorder)
	
	xiv	Drug Abuse Screening Test (DAST) [69]*	20 items; yes/no questions; Cronbach alpha: 0.74
	
	xv	Alcohol Use Disorders Identification Test (AUDIT) [70]*	10 items; 2 or multiple choice questions; Cronbach alpha: 0.88
	
	xvi	Modified Observed Aggression Scale (MOAS) for Aggressive Behaviors [71]*	Assess 4 categories of aggressive behaviour: verbal aggression, aggression to propriety, self-inflicted aggression, physical aggression
	
	xvii	K-10 Psychological Distress Scale (K-10 PDS) [72]*	10 items; five-point Likert scale questions; area under the receiver operating characteristic curve of SMI: 0.854
	
	xviii	Barratt Impulsivity Scale (BIS) [73]*	30 items; four-point Likert scale questions

*: Measurement instruments validated in the French-speaking population

The dependent variable refers to 'individuals diagnosed with mental disorders in the previous 12 months who have used health services due to mental health problems.' Participants who were diagnosed as having at least one mental disorder were invited to identify services that they used to deal with their condition. Individuals had one of the following diagnoses: major depressive disorder, mania, social phobia, agoraphobia, panic disorder, post-traumatic stress disorder, or alcohol and drug dependence. Mental disorders were identified with the CCHS 1.2 version of the Composite International Diagnostic Interview (CIDI) [74], used in the Global Survey on Mental Health (WMH, 2000). This is a diagnostic tool that generates psychiatric diagnoses according to the definitions and criteria of ICD 10 and DSM IV. Alcohol and drug dependency were assessed using a short form of the CIDI (based upon the DSM-III-R criteria). Previous versions of the CIDI have demonstrated reliability and validity [75]. The questionnaire on the use of mental health services was also adapted from the CCHS 1.2. Health services refer to one of the following services or professionals: hospital bed, rehabilitation centre, psychologist, general practitioner, psychiatrist, case manager, toxicologist, nurse, social worker, psychotherapist, pharmacist, support group, telephone support, or other health professionals. At least one of the above consultations in the previous 12 months was considered as use of mental health services.

Predisposing factors included socio-demographic variables (age, gender, marital status, household composition and size, education, first language, country of birth), religious beliefs (importance attributed to spirituality and frequency of participation in religious activities), health beliefs (quality of life, self-perception of mental and physical health), knowledge (parental psychiatric history) and justice system (history of imprisonment). Enabling factors included variables associated with income (personal, household and main source), territory, social support, social stigmatisation and spatial variables of the social and built characteristics of the local residential area. A geographic information system was used to compute proximity to the closest health service, and measures of local area socio-economic statues (SES). Proximity measures were obtained by calculating the traveling distance between a participant's home and the closest health service, either using pedestrian roads or the motor vehicle network. Measures of area social composition, obtained from the 2006 Census at the dissemination area level, were re-compiled for each participant's local residential area using ego-centered circular buffers of 500 metres [76]. Such ego-centered methods, providing individualised measures of local areas, make it possible to use environmental predictors in classical regression models at the individual level. Measures of local area SES included the following: proportion of renters and recent movers, unemployment rate, active population level, household income and number of recent immigrants. Finally, needs-related factors included the number of mental disorders diagnosed in the 12 months preceding the interview: psychological distress, impulsiveness, and emotional problems as well as longstanding violence victimisation and longstanding aggressive behaviour.

### Analyses

Univariate, bivariate, and multivariate analyses were carried out. Univariate analyses entailed the calculation of the frequency distribution for categorical variables and mean values for continuous variables. In bivariate analyses, a single logistic regression was used to assess variables associated with health service utilisation in the 12 months prior to the interview, with the Alpha value set at P < 0.10. Variables that yielded a significant association in bivariate analyses were introduced into the multiple model through the application of the backward likelihood ratio elimination technique [77], with the Alpha value set at P < 0.05. The Hosmer-Lemeshow test was applied to assess the model goodness-of-fit and a Nagelkerke R square was generated to express the proportion of variance explained by the model.

## Results

Among the 406 individuals who have experienced at least one episode of mental health disorder in the 12 months preceding the interview and were selected for the analyses described below, 212 (52%) reported at least one occurrence of health service use.

The distribution characteristics of the 406 participants are displayed in Tables 3 and 4. The sample was 56% female. The mean age was 39 (SD: 13.1). Eighty-one percent were Canadian-born. Mental and physical health was deemed to be very good or excellent, respectively, by 24% and 27% of participants. Approximately half (51%) reported that they were satisfied or very satisfied with their lives. Almost half was employed (45%). Only 13% reported receiving social assistance. The mean household income was $43,650 CAD (SD: $38,179; Minimum: 0; Maximum: $228,000.00). The mean score for quality of life was 94.7 (SD: 18.2; Minimum: 26; Maximum: 131). The three mental health disorders most frequently reported were major depressive episode (52%), alcohol dependence (24%), and social phobia (20%). More than half (57%) of the participants reported having experienced emotional problems in the 12 months before the interview. Forty-nine percent declared being lifetime victims of violence, and 47% had a history of aggressive behaviour.

**Table 3:** Frequency distribution of participants with mental disorders in the 12 months before the interview (N = 406)

		Distribution of the entire sample of participants (N = 406)			Participants who have used health services (N = 212)		P value (used versus did not use health services)
			**n**	**%**	**n**	%*	

**Predisposing factors**	Gender	Female	229	56.4	134	58.5	.157
		Male	177	43.6	78	44.1	
	
	Country of birth	Canada	329	81.0	142	43.2	.001
		Other	68	15.1	32	47.1	
	
	Self-perception of mental health	Excellent/Very good	99	24.4	33	33.3	<.001
		Good	170	41.9	91	53.5	
		
		Fair/Poor	137	33.7	87	63.5	
	
	Self-perception of physical health	Excellent/Very good	111	27.4	50	45.0	<.001
		Good	145	35.7	74	51	
		
		Fair/Poor	150	36.9	88	58.7	
	
	General satisfaction with life	Very satisfied/Satisfied	208	51.3	97	46.6	<.001
		Neither satisfied nor dissatisfied	111	27.3	60	54.1	
		Dissatisfied/Very dissatisfied	83	20.4	53	63.9	

**Enabling factors**	Main source of income	Salary	183	45.1	99	54.1	.258
		Social welfare	52	12.8	25	48.1	.572
		Rent or retirement pension	19	4.7	8	42.1	.024
		Unemployment insurance	11	2.7	5	45.5	.258
		Other	17	4.1	9	56.3	.090

**Needs**	Emotional problems in the 12 months pre-interview		230	56.7	161	70.0	<.001
	Lifetime victim of violence		198	48.8	133	67.2	<.001
	Lifetime aggressive behaviour		191	47.0	117	61.3	<.001

	Types of mental health disorders in the 12 months pre-interview	Major depressive episode	209	51.5	129	61.7	<.001
		Alcohol dependence	97	23.9	43	44.3	<.001
		Social phobia	80	19.7	41	51.3	.001
		Drug dependence	77	19.0	34	44.2	<.001
		Mania	45	11.1	23	51.1	<.001
		Panic disorder	44	10.8	22	50.0	.002
		Agoraphobia	29	7.1	19	65.5	.017
		Post-traumatic stress disorder	18	4.4	8	44.4	.094

**Table 4:** Descriptive statistics of participants with mental disorders in the 12 months before the interview

		(N = 406)						
		**Distribution of the entire sample of participants (N = 406)**				**Participants who have used health services (N = 212)**		**P value**

		**Minimum**	**Maximum**	**Mean**	**SD**	**Mean**	**SD**	**P value**

**Predisposing factors**	Age	16	69	39.40	13.11	40.89	12.65	.088
**Enabling factors**	Quality of life SLDS score*	26	131	94.73	18.19	93.21	19.64	<.001
	Total household income	0	228000.00	43650.03	38179.43	43313.75	36478.15	.026
**Needs-related factors**	Psychological distress score	1.00	37.00	15.6755	7.75732	16.2786	7.65919	<.001
	Number of mental health disorders per subject	1	3	0.34	0.640	0.32	0.636	.760

**Health service utilisation**	Number of health services used in the previous 12 months	0.00	8.00	1.8715	1.38426	2.1	1.964	<.001

*Satisfaction with Life Domains Scale (SLDS) score varies from 20 to 140 - the least or worst possible score to the best score

** K-10 psychological distress scale (K-10 PDS) varies from 10 to 50 - the best possible score to the worst score

The distribution characteristics of the 212 participants who used health services are also displayed in Tables 3 and 4. Individuals who used health services were more often female and dissatisfied or highly dissatisfied with their quality of life; in addition, they deemed their mental and physical health to be fair or poor. These individuals also tended to be longstanding victims of violence or had a long history of aggressive behaviour. Compared with the sample of 406 as a whole, individuals who used health services also had more frequent emotional problems, serious depressive episodes or agoraphobia in the last 12 months.

### Multiple model

Variables significantly associated with service utilisation in bivariate analyses are displayed in Table 5. Bivariate linear analyses yielded five predisposing, four enabling, and three needs-related variables significantly associated with health service use.

**Table 5:** Variables associated with health service utilisation by individuals with mental health disorders (N = 406)

		Bivariate analyses (simple logistic regression)			Multiple regression logistic (backward LR elimination method)				
		**Beta**	**Sig.**	**OR**	**Beta**	**Sig.**	**OR**	**95% IC**	
								**Lower L.**	**Upper L.**

**Predisposing factors**	Gender (Male = 1)	-.576	.004	.562	-.705	.003	.494	.308	.792
	Age	.018	.017	1.019	.019	.039	1.019	1.001	1.038
	General satisfaction with life (= Very satisfied)	-.272	.006	.762					
	Self-perception of his physical health (= Excellent)	-.208	.028	.812					
	Self-perception of mental health (= Excellent)	-.446	.000	.640	-.303	.016	.739	.577	.946

**Enabling factors**	Quality of life score	-.010	.086	.990					
	Proportion of active population aged 15 and up	.000	.065	1.000					
	Proportion of rental accommodations	-.008	.026	.992	-.010	.013	.990	.982	.998
	Proportion of recent immigrants (1 year)	-.094	.094	.910					

**Needs**	Lifetime victim of violence	1.222	.000	3.393	.997	.000	2.710	1.698	4.327
	Lifetime aggressive behaviour	.678	.001	1.971					
	Emotional problems in the 12 months pre-interview	1.850	.000	6.358	1.532	.000	4.629	2.870	7.465
					Hosmer-Lemeshow test: Khi-square = 13.717; P = 0.089 Nagelkerke R^2^: 33.6%				

The multiple logistic regression model built using these variables and the backward elimination technique retained six variables. Variables associated with needs-related factors were the most strongly correlated variables in the model. Participants who reported emotional problems in the 12 months before the interview had a four-fold probability increase in health service use. Those who reported being longstanding victims of violence were twice as likely to use services. Among predisposing factors, perception of mental health, gender, and age emerged, with age as the most strongly associated variable in this category: older participants were more likely than younger participants to use services. Individuals who view their mental health as poor were 73% more likely to use services. Female participants were 49% more likely to use services. Finally, among enabling factors, only one variable was associated, negatively, with health service utilisation: proportion of rental housing. Individuals living in neighbourhoods where renters outnumber homeowners used fewer health services. The model's goodness-of-fit was very adequate, as shown by the Hosmer-Lemeshow test, with 34% variance expressed.

## Discussion

This study aimed to assess variables associated with health service utilisation by individuals diagnosed with mental disorders within a 12-month period in an urban setting in Montreal, Canada. Using the Andersen's behavioural model as a guide, a holistic set of variables categorised into the following domains was taken into consideration: predisposing, enabling, needs-related (independent variables), and service utilisation (dependent variable). To the most salient variables associated with health services utilisation according to the literature, we have added some novel variables (e.g., involvement in the justice system, neighbourhood and geospatial data), and hypothesised that some of these could also play a role in health services utilisation for mental health reasons.

Interestingly, roughly half of the 406 individuals who experienced a mental disorder in the past year self-rated their mental health as good and said they were highly satisfied with their life. Individuals who used health services had a worse perception of their mental health and lower life satisfaction, compared to individuals who didn't use health services. It is impossible to say, however, if the former truly have better mental health or if they underestimate their mental health problems.

Most studies have shown that about 33% of individuals with mental disorders used health services to treat their condition [78,79]. In our study, almost 50% of individuals diagnosed with mental disorders in the 12 past months did not seek professional help for their problems, despite universal healthcare access. It may be that most individuals used mental health services only when they believe that their disorders were severe, chronic or disabling or when they thought that professionals could significantly help them [80]. The proximity of a psychiatric hospital may explain the observed increased use of health services in the catchment area. Usually, individuals with mental disorders tend to live near their treatment centre. Furthermore, needs are more numerous in deprived urban areas [81,82]. Another reason why our study showed a higher number of users could be that, inversely to other studies [78,79], ours included only individuals aged between 15 and 65 years old. Individuals over 65 years are less likely to use services than middle-aged individuals [8,34,35].

In accordance with the Andersen's theory, needs-related factors were the prime predictors of health service utilisation in our study [14]. The close association between emotional problems and service use could be linked to the pain experienced by individuals who recognise their emotional problems, leading them to seek professional help. Emotional problems are often linked in studies with interpersonal relationships [83]. According to Caron and colleagues, lack of emotional support and presence of persons perceived as stressful are the most significant predictors of psychological distress [84]. Some studies found an association between marital distress and mental health service use [83,85,86]. While marital concord is consistent with healthy sleep habits, fewer signs of depression, and fewer visits to the doctor's office, conjugal discord raises the risk of mental and physical dysfunction [86]. The loss of a relationship and the resulting social isolation has been shown to increase the use of health services by those who are separated, divorced or widowed [8,40,41].

With regard to longstanding victims of violence, several studies have revealed that exposure to violence intensifies stress and gives rise to feelings of vulnerability, thereby increasing the risk of developing mental disorders [52,87,88], and the subjective sense of having emotional problems [54]. Females who are victims both of physical and sexual violence are up to 14 times more likely to develop post-traumatic stress disorder, and five to eight times more likely to experience a serious depressive episode than females who have not been exposed to violence [87]. Furthermore, having a mental disorder increases the risk of exposure to violence. Victimisation is especially prevalent among individuals with serious mental disorders. Depending on the type of violent crime, the risk of the latter being a crime victim is six to twenty-three times greater than among the general population [88]. Psychiatric surveys do not usually show the percentage of individuals who are lifetime victims of violence; consequently, that variable is not analysed in studies on service use with respect to mental disorders. It wasn't possible to ascertain, however, if victims of violence were over represented in our sample.

With regards to predisposing factors, our results were consistent with previous findings that females were more likely to use health services [2,29,36,38]. However, women's greater propensity for using health services is not associated with a greater prevalence of mental disorder among them. In Quebec, the suicide rate among men is nearly four times greater than among women; in addition, substance-related disorders are significantly more frequent among men [20]. According to several authors, females have a greater facility for identifying and accepting mental health diagnoses, which may account for their more frequent use of mental health services [7,38].

The association between age and health service utilisation has been frequently noted in the literature [2,8,29,35,89]. In our sample, the mean age was roundly 39 years old (SD 13.1); participants were thus predominantly middle-aged. According to studies, these individuals were more likely to use services than those under 25 and those over 65 [8,34,35]. Younger adults tend not to perceive their need for treatment, and often prefer to solve problems by themselves [35]. They are also more likely to drop out of treatment [89]. Conversely, older individuals with mental disorders usually present more stable conditions, which may explain their preference for general practitioners as the main providers of health services [29]. The fear of stigmatisation may also contribute to lower health service use among young adults, and persons aged over 65 years old as compared with individuals in between [90].

The association between poor self-perception of mental health and health service use is evident. The failure to recognise a problem is a serious obstacle to health service access [49]. Accepting the need for mental health may be a challenge for individuals who are shy or who have high self-esteem [49]. Self-perception with regard to mental health or attitudes toward health services may account for differences between males and females [80]. Males are usually more reluctant to recognise their emotional or mental problems [80] and take longer to seek help [7,37].

The only enabling variable associated with health service use within a neighbourhood was the ratio of renters to homeowners. Neighbourhoods with more rental housing may be characterised by weaker residential stability. Conversely, homeowners may feel a greater sense of belonging to their neighbourhood. As they are less likely to move than renters, homeowners probably possess a better understanding of health services in their neighbourhood and may even have used them regularly for a long period. Moreover, residential stability fosters the development of stronger ties with neighbours that can help individuals to identify their problems and determine what to do to resolve them [49,52]. Perhaps, neighbourhoods with a larger proportion of homeowners also enjoy higher per-capita income. Individuals with higher socio-economic status have access to a greater range of resources [2], such as private psychologists whose services are not covered by the public healthcare insurance system in Quebec.

## Limitations

This study has some limitations. First, it did not include the full spectrum of psychiatric disorders, for example, schizophrenia and other serious mental disorders, organic mental disorders, sexual disorders, eating disorders, personality disorders or intellectual deficiencies. Individuals with severe mental disorders have been heavy service users [91-93]. Second, we did not take into account the severity of mental disorder. Previous studies have reported that severe cases were associated with more intensive service use than mildly severe or moderate cases [11,12]. Third, we didn't have information about the duration of, or access to health service use. According to Leaf and colleagues, health service use is associated with having a regular source of medical care [34]. Long delays between the onset of disease and first contact with a health provider represent a significant obstacle to health service use [7]. Fourth, the observed correlations between health service use and the diverse variables didn't imply causality. Finally, the value of this study may be limited to information regarding the specific city or the specific catchment area that was investigated.

## Conclusion

The present study is of interest as it analysed a comprehensive set of variables, including geospatial factors. To our knowledge, no previous study has focused on such numerous variables using the Andersen behavioural model. This catchment area study also made an original contribution by targeting individuals diagnosed with mental disorders exclusively as against much of the literature which focuses on the general population.

As reported in previous studies, an unusually high proportion of individuals with mental disorders did not use health services. If decision-makers and practitioners are to improve the efficacy of the mental health system, they must gain a better understanding of factors that drive the utilisation or non-utilisation of services. This study broke new ground by uncovering the importance of two novels variables (longstanding violence victimisation, and the proportion of homeownership), associated with health service use among individuals with mental disorders. Stress and pain caused by needs-related factor or health belief variable (longstanding violence victimisation, emotional problems, self-perceived mental health) may lead individuals to seek health services for mental health reasons. In addition, targeting neighbourhoods with high proportion of rental accommodations could be a public health priority to improve service utilization and mental health. Finally, as reported in previous research, age and gender were associated with health service use. Males and youths used significantly fewer mental health services than females and middle-aged individuals. Accordingly, strategies aimed at changing attitudes among males and youths should be promoted. Globally, this study encourages the reinforcement of public education, and services designed especially to target populations (youths and males, rental housing neighbourhoods), in view of helping individuals with mental disorders or reducing their reluctance to seek health services.

## Competing interests

The authors declare that they have no competing interests.

## Authors' contributions

MJF, GG and MP designed the study. JMB carried out the statistical analyses with assistance from JC directing the full catchment area study. MJF and GG wrote the article. YK built the geospatial data-bank used in the study, and helped to write the methodological section. All authors have read and approved the final manuscript.

## References

[B1] Dewa CS Lesage A Goering P Craven M Nature and prevalence of mental illness in the workplace Healthc Pap 2004 5 2 12 25 15829761 10.12927/hcpap..16820

[B2] Vasiliadis HM Lesage A Adair C Wang PS Kessler RC Do Canada and the United States differ in prevalence of depression and utilization of services Psychiatr Serv 2007 58 1 63 71 10.1176/appi.ps.58.1.63 17215414

[B3] Wang PS Beck AL Berglund P McKenas DK Pronk NP Simon GE Kessler RC Effects of major depression on moment-in-time work performance Am J Psychiatry 2004 161 10 1885 1891 10.1176/appi.ajp.161.10.1885 15465987

[B4] Kessler RC Berglund P Demler O Jin R Koretz D Merikangas KR Rush AJ Walters EE Wang P Replication NCS. The epidemiology of major depressive disorder. Results from the National Comorbidity Survey Replication (NCS-R) JAMA 2003 289 23 3095 3105 10.1001/jama.289.23.3095 12813115

[B5] Mojtabai R Olfson M Mechanic D Perceived need and help-seeking in adults with mood, anxiety, or substance use disorders Arch Gen Psychiatry 2002 59 1 77 84 10.1001/archpsyc.59.1.77 11779286

[B6] Kessler RC Zhao S Katz SJ Kouzis AC Frank RG Edlund M Leaf P Past-year use of outpatient services for psychiatric problems in the National Comorbidity Survey Am J Psychiatry 1999 156 1 115 123 9892306 10.1176/ajp.156.1.115

[B7] Wang PS Berglund P Olfson M Pincus HA Wells KB Kessler RC Failure and delay in initial treatment contact after first onset of mental disorders in the National Comorbidity Survey Replication Arch Gen Psychiatry 2005 62 6 603 613 10.1001/archpsyc.62.6.603 15939838

[B8] Wang PS Lane M Olfson M Pincus HA Wells KB Kessler RC Twelve-month use of mental health services in the United States Arch Gen Psychiatry 2005 62 6 629 640 10.1001/archpsyc.62.6.629 15939840

[B9] Vasiliadis H-M Lesage A Adair C Boyer R Service use for mental health raisons: cross-provincial difference in rates, determinants, and equity of access Can J Psychiatry 2005 50 10 614 619 16276852 10.1177/070674370505001007

[B10] Wittchen H-U Jacobi F Size and burden of mental disorders in Europe - a critical review and appraisal of 27 studies Eur Neuropsychopharmacol 2005 15 4 357 376 10.1016/j.euroneuro.2005.04.012 15961293

[B11] Tempier R Meadows GN Vasiliadis HM Mosier KE Lesage A Stiller A Graham A Lepnurm M Mental disorders and mental health care in Canada and Australia: comparative epidemiological findings Soc Psychiatry Psychiatr Epidemiol 2009 44 1 63 72 10.1007/s00127-008-0409-y 18626555

[B12] Wang PS Aguilar-Gaxiola S Alonso J Angermeyer MC Borges G Bromet EJ Bruffaerts R de Girolama G de Graff R Gureje O Use of mental health services for anxiety, mood, and substance disorders in 17 countries in the WHO world mental health surveys Lancet 2007 370 9590 841 850 10.1016/S0140-6736(07)61414-7 17826169 PMC2847360

[B13] Lesage A Vasiliadis H-M Gagné M-A Dudgeon S Kasman N Hay C Prevalence of mental illness and related service utilization in Canada: an analysis of the Canadian Community Health Survey 2006 Mississauga, Ontario: Canadian Collaborative Mental Health Initiative

[B14] Andersen RM Revisiting the behavioral model and access to medical care: does it matter? J Health Soc Behav 1995 36 1 1 10 10.2307/2137284 7738325

[B15] Tsao JC Dobalian A Myers CD Zeltzer LK Pain and use of complementary and alternative medicine in a national sample of persons living with HIV J Pain Symptom Manage 2005 30 5 418 432 10.1016/j.jpainsymman.2005.05.012 16310616 PMC1420635

[B16] Digiusto E Treloar C Equity of access to treatment, and barriers to treatment for illicit drug use in Australia Addiction 2007 102 6 958 969 10.1111/j.1360-0443.2007.01842.x 17523991

[B17] Vingilis E Wade T Seeley J Predictors of adolescent health care utilization J Adolesc 2007 30 5 773 800 10.1016/j.adolescence.2006.10.001 17141307

[B18] Chou YC Lee YC Lin LC Chang AN Huang WY Social services utilization by adults with intellectual disabilities and their families Soc Sci Med 2008 66 12 2474 2485 18339467 10.1016/j.socscimed.2008.01.055

[B19] Lin E Goering PN Lesage A Streiner DL Epidemiologic assessment of overmet need in mental health care Soc Psychiatry Psychiatr Epidemiol 1997 32 6 355 362 10.1007/BF00805441 9299930

[B20] Lefebvre J Lesage A Cyr M Toupin J Fournier L Factors related to utilization of services for mental health reasons in Montreal, Canada Soc Psychiatry Psychiatr Epidemiol 1998 33 6 291 298 10.1007/s001270050057 9640098

[B21] Bijl RV Ravelli A Psychiatric morbidity, service use, and need for care in the general population: results of the netherlands mental health survey and incidence study Am J Public Health 2000 90 4 602 607 10754976 10.2105/ajph.90.4.602 PMC1446190

[B22] Goodwin R Andersen RM Use of the behavioral model of health care use to identify correlates of use of treatment for panic attacks in the community Soc Psychiatry Psychiatr Epidemiol 2002 37 5 212 219 10.1007/s00127-002-0543-x 12107712

[B23] Elhai JD Grubaugh AL Richardson JD Egede LE Creamer M Outpatient medical and mental healthcare utilization models among military veterans: Results from the 2001 National Surveys of Veterans J Psychiatr Research 2008 42 10 858 867 10.1016/j.jpsychires.2007.09.006 18005993

[B24] Thorpe JM Van Houtven CJ Sleath BL Thorpe CT Rural-urban differences in preventable hospitalizations among community-dwelling veterans with dementia J Rural Health 2010 26 2 146 155 10.1111/j.1748-0361.2010.00276.x 20447001 PMC3691102

[B25] Choi S Rozario P Morrow-Howell N Proctor E Elders with first psychiatric hospitalization for depression Int J Geriatr Psychiatry 2009 24 1 33 40 10.1002/gps.2064 18543349 PMC2605168

[B26] Kamble P Chen H Sherer J Aparasu RR Antipsychotic drug use among elderly nursing home residents with dementia in the United States Am J Geriatr Pharmacother 2009 6 4 187 197 19028374 10.1016/j.amjopharm.2008.10.002

[B27] Fleury M-J Grenier G Bamvita J-M Caron J Professional service utilisation among patients with severe mental disorders BMC health services research 2010 10 141 20507597 10.1186/1472-6963-10-141 PMC2896947

[B28] Fleury M-J Grenier G Bamvita J-M Caron J Mental health service utilization among patients with severe mental disorders Community Ment Health J 2010 47 4 365 377 20490675 10.1007/s10597-010-9320-6

[B29] Carr VJ Johnston PJ Lewin TJ Rajkumar S Carter GL Issakidis C Patterns of service use among persons with schizophrenia and other psychotic disorders Psychiatr Serv 2003 54 2 226 235 10.1176/appi.ps.54.2.226 12556605

[B30] Hatzenbuehler ML Keyes KM Narrow WE Grant BF Hasin DS Racial/ethnic disparities in service utilization for individuals with co-occurring mental health and substance use disorders in the general population J Clin Psychiatry 2008 69 7 1112 1121 10.4088/JCP.v69n0711 18517286 PMC2745048

[B31] Lemming MR Calsyn RJ Utility of the behavioral model in predicting service utilization by individuals suffering from severe mental illness and homelessness Community Ment Health J 2004 40 4 347 364 15453086 10.1023/b:comh.0000035229.20557.5c

[B32] Lipsky S Caetano R Roy-Byrne P Triple jeopardy; impact of partner violence perpetration, mental health and substance abuse on perceived unmet for mental health care among men Soc Psychiatry Psychiatr Epidemiol 2010 46 9 843 852 20582398 10.1007/s00127-010-0258-3 PMC3010419

[B33] Keyes KM Hatzenbuehler ML Alberti P Narrow WE Grant BF Hasin DS Service utilization differences for Axis I psychiatric and substance use disorders between white and black adults Psychiatr Serv 2008 59 8 893 901 10.1176/appi.ps.59.8.893 18678687 PMC2729457

[B34] Leaf PJ Livingston MM Tischler GL Weissman MM Holzer CE Myers JK Contact with health professionals for the treatment of psychiatric and emotional problems Med Care 1985 23 12 1322 1337 10.1097/00005650-198512000-00002 4087948

[B35] Kessler RC Berglund PA Bruce ML Koch JR Laska EM Leaf PJ Mandercheid RW Rosenheck RA Walters EE Wang PS The prevalence and correlates of untreated serious mental illness Health Serv Res 2001 36 6 987 1007 11775672 PMC1089274

[B36] Narrow WE Regier DA Norquist G Rae DS Kennedy C Arons B Mental health service use by Americans with severe mental illness Soc Psychiatry Psychiatr Epidemiol 2000 35 4 147 155 10.1007/s001270050197 10868079

[B37] Putkonen H Weizman-Henelius G Lindberg N Romano T Häkkänen-Nyholm H Gender differences in homicide offenders' criminal career, substance abuse and mental health care. A nationwide register-based study of Finnish homicide offender 1995-2004 Crim Behav Ment Health 2011 21 1 51 62 20603817 10.1002/cbm.782

[B38] Uebelacker LA Wang PS Berglund P Kessler RC Clinical differences among patients treated for mental health problems in general medical and specialty mental health settings in the National Comoribidity Survey Replication (NCS-R) Gen Hosp Psychiatry 2006 28 5 387 395 10.1016/j.genhosppsych.2006.05.001 16950373 PMC2694036

[B39] Wells KB Manning WG Duan N Newhouse JP Ware JE Jr Sociodemographic factors and the use of outpatient mental health services Med care 1986 24 1 75 85 10.1097/00005650-198601000-00008 3945131

[B40] Bebbington P Meltzer H Brugha TS Farrell M Jenkins R Ceresa C Lewis G Unequal access and unmet need: neurotic disorders and the use of primary care services Psychol Med 2000 30 6 1359 1367 10.1017/S0033291799002950 11097076

[B41] Parslow RA Jorm AF Who uses mental health services in Australia? An analysis of data from the National Survey of Mental Health and Wellbeing Aust N Z J Psychiatry 2000 34 6 997 1008 10.1080/000486700276 11127632

[B42] Olfson M Marcus SC Druss B Pincus HA National trends in the use of outpatient psychotherapy Am J Psychiatry 2002 159 11 1914 1920 10.1176/appi.ajp.159.11.1914 12411228

[B43] Young AS Klap R Sherbourne CD Wells KB The quality of care for depressive and anxiety disorders in the United States Arch Gen Psychiatry 2001 58 1 55 61 10.1001/archpsyc.58.1.55 11146758

[B44] Wang PS Berglund P Kessler RC Recent care of common mental disorders in the United States: prevalence and conformance with evidence-based recommendations J Gen Intern Med 2000 15 5 284 292 10.1046/j.1525-1497.2000.9908044.x 10840263 PMC1495452

[B45] Hendryx MS Ahern MM Acess to mental health services and health sector social capital Adm Policy Ment Health 2001 28 3 205 217 10.1023/A:1007860002137 11330016

[B46] Alegria M Bijl RV Lin E Walters EE Kessler RC Income differences in persons seeking outpatient treatment for mental disorders Arch Gen Psychiatry 2000 57 4 383 391 10.1001/archpsyc.57.4.383 10768701

[B47] Pescosolido BA Gardner CB Lubell KM How people get into mental health services: stories of choice, coercion and "muddling through" from "first-timers" Soc Sci Med 1998 46 2 275 286 10.1016/S0277-9536(97)00160-3 9447648

[B48] Albert M Becker T McCrone P Thornicroft G Social networks and mental health service utilisation. A literature review Int J Soc Psychiatry 1998 44 4 248 266 10.1177/002076409804400402 10459509

[B49] Howard KI Cornille TA Lyons JS Vessey JT Lueger RJ Saunders SM Patterns of mental health service utilization Arch Gen Psychiatry 1996 53 8 696 703 10.1001/archpsyc.1996.01830080048009 8694683

[B50] Bonin J-P Fournier L Blais R Predictors of mental health service utilization by people using resources for homeless people in Canada Psychiatr Serv 2007 58 7 936 941 10.1176/appi.ps.58.7.936 17602009

[B51] Galea S Ahern J Rudenstine S Wallace Z Vlahow D Urban built environment and depression: a multilevel analysis J Epidemiol Comnmunity Health 2005 59 10 822 827 10.1136/jech.2005.033084 PMC1732922 16166352

[B52] Stockdale SE Wells KB Tang L Belin TR Zhang L Sherbroune CD The importance of social context; neighborhood stressors, stress-buffering mechanisms, and alcohol drug, and mental health disorders Soc Sci Med 2007 65 9 1867 1881 10.1016/j.socscimed.2007.05.045 17614176 PMC2151971

[B53] Amstadter A Zinzow HM McCauley JL Strachan M Ruggerio KJ Resnick HS Kilpatrick DG Prevalence and correlates of service utilization and help seeking in a national college sample of female rape victims J Anxiety Disord 2010 24 8 900 902 10.1016/j.janxdis.2010.06.014 20620018 PMC3687342

[B54] Brand B Trauma and women Psychiatr Clin North Am 2003 26 3 759 579 10.1016/S0193-953X(03)00034-0 14563108

[B55] Caron J Tousignant M Pedersen D Fleury M-J Cargo M Daniel M Kestin Y Crocker A Perreault M Brunet A La création d'une nouvelle génération d'études épidémiologiques en santé mentale Sante Ment Que 2007 32 2 225 238

[B56] Singer E Nonresponse bias in household surveys Public Opin Q 2006 70 5 637 645 10.1093/poq/nfl034

[B57] Morton LM Cahill J Hartge P Reporting participation in epidemiologic studies: a survey of practice Am J Epdemiol 2006 163 3 197 203 10.1093/aje/kwj036 16339049

[B58] NPHS National Public health Survey (NPHS) Ottawa: Statistics Canada 2003 2005

[B59] Baker F Intagliata J Quality of life in the evaluation of community support systems Eval Program Plann 1982 5 1 69 79 10.1016/0149-7189(82)90059-3 10257372

[B60] Driessen G Gunther N Van Os J Shared social environment and psychiatric disorder: a multilevel analysis of individual and ecological effects Soc Psychiatry Psychiatr Epidemiol 1998 33 12 606 612 10.1007/s001270050100 9857793

[B61] Perkins DD Long DA Fisher AT, Sonn CC, Bishop BJ Neighborhoud sense of community and social capital: A multi-level analysis Psychological sense of community: Research, applications and implications 2002 New York: Plenum 291 318

[B62] Saegert S Winke G Crime, social capital, and community participation Am J Community Psychol 2004 34 3-4 219 233 10.1007/s10464-004-7416-2 15663208

[B63] Nario-Redmond MR Coulton CJ Milligan SE Measuring resident perceptions of neighborhood conditions: Survey methodology 2000 Cleveland, Ohio: Case Western Reserve University, Mandel School of Applied Social Sciences, Center on Urban Poverty and Social Change

[B64] Sampson RJ Morenoff JD Gannon-Rowley T Assessing neighborhood effects: social processes and new directions in research Annu Rev Soc 2002 28 443 478 10.1146/annurev.soc.28.110601.141114

[B65] Coulton CJ Korbin JE Su M Measuring neighborhood context for young children in an urban area Am J Community Psychol 1996 24 1 5 32 10.1007/BF02511881

[B66] Cutrona CE Behavioral manifestation of social support: a microanalytic investigation J Pers Soc Psychol 1986 51 1 201 208 3735068 10.1037/0022-3514.51.1.201

[B67] Tohen M Bromet E Murphy JM Tsuang MT Psychiatric epidemiology Harv Rev Psychiatry 2000 8 3 111 125 10973936

[B68] Andrade L Caraveo-Anduaga JJ Berglund P Bijl RV Kessler RC Demler O Walters EE Kylyc C Offord D Üstün TB Cross-national comparisons of the prevalences and correlates of mental disorders Bulletin World Health Organization 2000 78 4 421 426 PMC2560724 10885160

[B69] Skinner HA The Drug Abuse Screening Test Addictive Behaviors 1982 7 4 363 371 10.1016/0306-4603(82)90005-3 7183189

[B70] Bohn MJ Babor TF Kranzler HR The Alcohol Use Disorders Identification Test (AUDIT): validation of a screening instrument for use in medial settings J Stud Alcohol 1995 56 4 423 432 7674678 10.15288/jsa.1995.56.423

[B71] Kay SR Woldkenfied F Murrill LM Profiles of aggression among psychiatrist patients J Nerv Ment Dis 1988 176 9 539 546 10.1097/00005053-198809000-00007 3418327

[B72] Kessler RC Barker PR Colpe LJ Epstein JF Gfroerer JC Hiripi E Howes MJ Normand S-L Mandercheid RW Walters EE Screening for serious mental illness in the general population Arch Gen Psychiatry 2003 60 2 184 189 10.1001/archpsyc.60.2.184 12578436

[B73] Barratt ES Spence JT, Izards CE Impulsiveness subtraits: Arousal and information processing Motivation, emotion and personality 1985 North Holland: Elsevier Science Publishers 137 146

[B74] Kessler RC Andrews G Mroczek D Ustun B Wittchen HU The World Health Organization Composite International Diagnostic Interview Short From (CIDI-SF) Int J Methods Psychiatr Res 1998 7 4 171 185 10.1002/mpr.47

[B75] Wittchen HU Reliability and validity studies of the WHO-Composite International Diagnostic Interview (CIDI): a critical review J Psychiatr Res 1994 28 1 57 84 10.1016/0022-3956(94)90036-1 8064641

[B76] Oliver LN Schuurman N Hall AW Comparing circular and network buffers to examine the influence of land use on walking for leisure and errands Int J Health Geographics 2007 6 41 10.1186/1476-072X-6-41 PMC2034381 17883870

[B77] Field A Discovering statistics using SPSS 2005 2 London: Sage

[B78] Kessler RC Demler O Frank RG Olfson M Pincus HA Walters EE Wang PS Wells KB Zaslavsky AM Prevalence and treatment of mental disorders, 1990 to 2003 N Engl J Med 2005 352 24 2515 2523 15958807 10.1056/NEJMsa043266 PMC2847367

[B79] Andrews G Henderson S Hall W Prevalence, comorbidity, disability and service Br J Psychiatry 2001 178 145 153 10.1192/bjp.178.2.145 11157427

[B80] Andrews G Issakidis C Carter G Shortfall in mental health service utilisation British Journal of Psychiatry 2001 179 417 425 10.1192/bjp.179.5.417 11689399

[B81] Najim H McCrone P The Camberwell Assessment of Need: comparison of assessments by staff and patients in an inner-city and a semi-rural community area Psychiatr Bull 2005 29 1 13 17 10.1192/pb.29.1.13

[B82] McCrone P Leese M Thornicroft G Griffith G Padfield S Schene AH Knudsen HC Vazquez-Barquero JL Lasalvia A White IR Reliability of the Camberwell Assessment of Need- European Version. EPSILON Study 6. European Psychiatric Services: Inputs inked to outcome domains and needs Br J Psychiatry Suppl 2000 39 s34 s40 10945076 10.1192/bjp.177.39.s34

[B83] Whisman MA Uebelacker LA Impairment and distress associated with relationship discord in a national sample of married or cohabiting adults J Fam Psychol 2006 20 3 369 377 16937993 10.1037/0893-3200.20.3.369

[B84] Caron J Latimer E Tousignant M Predictors of psychological Distress in Low-income populations of Montreal Can J Public Health 2007 98 1 s35 s44 18047159 10.1007/BF03403725 PMC6975867

[B85] Schonbrun YC Whismam MA Marital distress and mental health care service utilization J Consult Clin Psychol 2010 78 5 732 736 20873908 10.1037/a0019711

[B86] Prigerson HG Maciejewski PK Rosenheck RA The effects of marital dissolution and marital quality on health and health service use among women Med Care 1999 37 9 858 873 10.1097/00005650-199909000-00003 10493465

[B87] Hedtke KA Ruggiero KJ Fitzerald MM Zinzow HM Saunders BE Resnick HS Kilpatrick DG A longitudinal investigation of interpersonal violence in relation to mental health and substance use J Consult Clin Psychol 2008 76 4 633 647 18665691 10.1037/0022-006X.76.4.633

[B88] Teplin LA McClelland GM Abram KM Weiner DA Crime victimization in adults with severe mental illness: comparison with the National Crime Victimization Survey Arch Gen Psychiatry 2005 62 8 911 921 16061769 10.1001/archpsyc.62.8.911 PMC1389236

[B89] Edlund MJ Wang PS Berglund PA Katz SJ Lin E Kessler RC Dropping out of mental health treatment: patterns and predictors among epidemiological survey respondents in the United States and Ontario Am J Psychiatry 2002 159 5 845 851 10.1176/appi.ajp.159.5.845 11986140

[B90] Kessler RC Chiu WT Demler O Walters EE Prevalence, severity, and comorbidity of 12-month DSM-IV disorders in the National Comorbidity Survey Replication Arch Gen Psychiatry 2005 62 6 617 627 10.1001/archpsyc.62.6.617 15939839 PMC2847357

[B91] Kent S Fogarty M Yellowlees P A review of studies of heavy users of psychiatric services Psychiatr Serv 1995 46 12 1247 1253 8590109 10.1176/ps.46.12.1247

[B92] Kent S Yellowlees P The relationship between social factors and frequent use of psychiatric services Aust N Z Psychiatry 1995 29 3 403 408 10.3109/00048679509064947 8573042

[B93] Chaput YJ Lebel M-J Demographic and clinical profiles of patients who make multiple visits to psychiatric emergency services Psychiatr Serv 2007 58 3 335 341 10.1176/appi.ps.58.3.335 17325106

